# Liquid Phase Sintering of (Ti,Zr)C with WC-Co

**DOI:** 10.3390/ma10010057

**Published:** 2017-01-11

**Authors:** Taoran Ma, Rafael Borrajo-Pelaez, Peter Hedström, Andreas Blomqvist, Ida Borgh, Susanne Norgren, Joakim Odqvist

**Affiliations:** 1 Department of Materials Science and Engineering, KTH Royal Institute of Technology, Stockholm SE-100 44, Sweden; rafaelbp@kth.se (R.B.-P.); pheds@kth.se (P.H.); odqvist@kth.se (J.O.); 2 Sandvik Coromant Research and Development, Stockholm SE-126 80, Sweden; andreas.blomqvist@sandvik.com (A.B.); susanne.m.norgren@sandvik.com (S.N.); 3 Sandvik Mining AB, Research and Development Rock Tools, Stockholm SE-126 80, Sweden; ida.borgh@sandvik.com

**Keywords:** cemented carbides, ternary cubic carbide, liquid-phase sintering, scanning electron microscopy, energy dispersive X-ray spectroscopy, dilatometer, differential scanning calorimetry

## Abstract

(Ti,Zr)C powder was sintered with WC-Co following an industrial process, including an isotherm at 1410 °C. A series of interrupted sintering trials was performed with the aim of studying the sintering behavior and the microstructural evolution during both solid-state and liquid-state sintering. Reference samples, using the same elemental compositions but with the starting components TiC and ZrC instead of (Ti,Zr)C, were also sintered. The microstructure was investigated using scanning electron microscopy and energy dispersive X-ray spectroscopy. It is found that the (Ti,Zr)C phase decomposes into Ti-rich and Zr-rich nano-scale lamellae before the liquid-state of the sintering initiates. The final microstructure consists of the binder and WC as well as two different γ phases, rich in either Ti (γ_1_) or Zr (γ_2_). The γ_2_ phase grains have a core-shell structure with a (Ti,Zr)C core following the full sintering cycle. The major differences observed in (Ti,Zr)C with respect to the reference samples after the full sintering cycle were the referred core-shell structure and the carbide grain sizes; additionally, the microstructural evolution during sintering differs. The grain size of carbides (WC, γ_1_, and γ_2_) is about 10% smaller in WC-(Ti,Zr)C-Co than WC-TiC-ZrC-Co. The shrinkage behavior and hardness of both composites are reported and discussed.

## 1. Introduction

Cemented carbides are often used in tools for metal cutting. Traditionally they are comprised of a hard phase (WC) and a ductile phase (Co), but in order to improve hardness and wear resistance, one or several hard transition metal carbides or carbonitrides, e.g., TiC, VC, TaC and Ti(C,N), can be added. These additional cubic phases are commonly called γ-phases.

Cemented carbide tools for metal cutting are manufactured by liquid-phase sintering, and it is crucial to control the average size and size distribution of WC grains, since they affect the final mechanical properties of the tool [1,2]. It is well-known that the addition (often by adding a γ-phase) of certain elements, such as Ti, Cr and V, could effectively inhibit WC grain growth during sintering [3,4,5]. Another example of the favorable utilization of a γ-phase is in gradient-sintered cemented carbide, during which the addition of complex carbides or carbonitrides solid solution, e.g., (Ti,W)(C,N), (Zr,W)C, and (Zr,Nb)C, can give rise to a tough zone close to the cutting edge, either after sintering or by post-sintering heat-treatment in a controlled atmosphere [6,7,8,9]. However, the potential benefits, in terms of cemented carbide performance, associated with phase transformation within the γ-phase during sintering have not been explored to the same extent.

When discussing materials for hard coatings, Holleck [10,11] reported results where ternary mixed carbides, e.g., (V,Ta)C and (Ti,Hf)C were substantially harder than their binary carbide constituents. Since both the pseudo-binary systems VC-TaC and TiC-HfC have miscibility gaps, one possible explanation for the increased hardness is that it results from a decomposition of the mixed carbide inside the miscibility gap. This finding inspired Borgh et al. [12,13] to study the decomposition of the mixed carbide (Ti,Zr)C during aging inside the miscibility gap. Ma et al. [14] later performed a nanoindentation study of the decomposed (Ti,Zr)C and found that it was superhard, at around 40 GPa. Therefore, the mixed carbide (Ti,Zr)C appears to be an interesting choice as a γ-phase in a WC-Co alloy, considering that the mixed carbide and the decomposed variants have beneficial mechanical properties. Furthermore, it is interesting to consider whether, and how, the (Ti,Zr)C will decompose in the presence of WC and Co.

There are a limited number of previous studies on the sintering of the W-Ti-Zr-Co-C system available in the literature. Markström and Frisk [15] studied the miscibility gap in TiC-ZrC and Ti(C,N)-Zr(C,N) in equilibrium with WC-Co, and Borgh et al. [16] studied the influence of the N_2_ partial pressure on the miscibility gap in the (Ti,Zr,W)(C,N) system, which was sintered with WC-Co. Weidow et al. [17] demonstrated that the addition of both TiC and ZrC powders appears to be effective in inhibiting grain growth, resulting in reduced carbide grain size and elevated hardness. In the aforementioned studies, the material was prepared from individual TiC and ZrC powders, and hence they did not investigate a possible demixing behavior. On the other hand, Xu et al. [18] performed sintering trials starting with a single phase W-Ti-Zr-C powder mixed with Co and suggested that the initial phase (Ti,Zr,W)C decomposed via spinodal decomposition in materials starting with 20 WC-40 TiC-40 ZrC (in mol %). This decomposition was characterized based on the side bands that were observed in the X-ray diffraction patterns. Furthermore, the alloys with decomposition were found to be about 20% harder than those without decomposition. Hall et al. [19] claimed that a spinodally decomposed structure was produced after sintering (Ti,Zr)C powder with WC-Co, which was fine-grained and showed high resistance to abrasion. However, no microscopy evidence for a decomposed microstructure was provided to support their claims.

The purpose of the present work is to study the sintering behavior of WC-(Ti,Zr)C-Co and the microstructural evolution during both the solid-state and the final liquid-state sintering. Composite samples were prepared from either mixed (Ti,Zr)C or individual TiC and ZrC powders. A series of interrupted sintering trials was performed, and the resulting microstructure was studied using scanning electron microscopy (SEM) and energy dispersive X-ray spectroscopy (EDS). In addition, the shrinkage behavior, melting, and mechanical properties were also investigated using dilatometry, differential scanning calorimetry (DSC), and Vickers hardness, respectively.

## 2. Experimental Procedures

### 2.1. Sintering Trials

Two cemented carbide composites were studied in the present work: composite 1 is WC-(Ti,Zr)C-Co, which utilizes a (Ti,Zr)C powder as the initial cubic carbide; composite 2 is WC-TiC-ZrC-Co produced from individual TiC and ZrC powders, and it is used as a reference sample. Both composites were manufactured with equal elemental compositions, i.e., Ti (2.9 wt %), Zr (5.2 wt %) W (75.5 wt %), Co (10 wt %), and a balance of C. The WC powder used in the present study was manufactured by Wolfram Bergbau und Hütten AG (Mittersill, Austria); the particle size was 5.8 µm, and the purity was 99.99 wt %. The Co powder was manufactured by Umicore (Brussels, Belgium); the particle size was 1.4 µm, and the purity was 99.76 wt %. The TiC powder was manufactured by Treibacher Industrie AG (Althofen, Austria); the particle size was 1.5 µm, and the purity was 99.6 wt %. The ZrC powders was manufactured by H.C. Starck (Newton, MA, USA); the particle size was 4.1 µm, and the purity was 97.2 wt %. The (Ti,Zr)C powder was synthesized by a carbothermal reduction of TiZrO_4_ at 2200 °C; the particle size was around 20 µm, and the average composition was Ti_0.5_Zr_0.5_C. Detailed information on the synthesization of the mixed carbide can be found in other work [13].

The powders were mixed with polyethylene glycol (PEG) in ethanol-water and milled in a 0.25 L rotating ball mill for 8 h at a rotating speed of 150 rpm, using WC-Co cylinders with a diameter of 7 mm as the milling body. The powders were then spray-dried and pressed into blanks. The green bodies were dewaxed and partly deoxidized at 450 °C for an isothermal period of 2 h, during which a flow of argon and hydrogen was supplied. Following this, the furnace was evacuated to form a vacuum, and the temperature was increased at a rate of 6 °C/min, until the isothermal sintering temperature of 1410 °C was reached. The samples were kept at that temperature for 1 h and then cooled at a controlled rate of 5 °C/min to 1250 °C, then 16 °C/min to 1000 °C, and were finally cooled to room temperature in the furnace by allowing it to self-cool.

With the purpose of tracking the microstructural evolution, interrupted sintering trials were performed at a series of temperatures between 1185 °C and 1400 °C in a DSC system Netzsch STA 409 CD (NETZSCH-Gerätebau GmbH, Selb, Germany) using a heating rate of 10 °C/min and an argon flow rate of 27 mL/min. The melting point of the composites was also measured. Furthermore, the shrinkage behavior of the composites was monitored using a Netzsch DIL 402C dilatometer (NETZSCH-Gerätebau GmbH, Selb, Germany). The composites were heated under vacuum conditions until they reach 1410 °C, with a heating rate of 10 °C/min. Prior to all the experiments performed with the dilatometer and DSC, the samples were dewaxed at 450 °C for 8 min under flowing argon and hydrogen. Thereafter, they were continuously heated in the desired atmosphere.

### 2.2. Characterization of Sintered Samples

The carbide grain size of the sintered samples was estimated from SEM images (magnification ×3000) using the linear intercept method, with 100 measurements on each γ phase and 160 measurements on the WC phase in each composite. The magnetic coercivity and the saturation of the samples were also measured. The coercivity provides indirect information on the grain size of the hard phase, with higher coercivity levels indicting a smaller grain size; whereas the magnetic saturation provides indirect information on the amount of tungsten dissolved in the binder phase. Vickers hardness was measured using a load of 3 kg. The porosity was determined in accordance with the ISO4505 standard for metallographic determination of porosity.

In order to characterize the phases present after sintering, X-ray diffraction (XRD) was performed with a Bruker D8 diffractometer (Cu-K_α_) equipped with a LynxEye 1D detector (Bruker AXS GmbH, Kalsruhe, Germany). The tube voltage and current were set to 40 kV and 40 mA, respectively. The 2-theta scan range used was 20° to 90°. The step size during the scans was 0.01°, and the acquisition time per step was 1 s.

The samples examined for microstructure characterization were mounted in a thermoset resin (KonductoMet^®^) and subsequently polished using 9 µm and 1 µm diamond suspension, and thereafter by using 0.02 µm colloidal silica. The final polishing was performed using a Hitachi IM4000 Broad-Beam Argon Ion milling system, (Hitachi High-Technologies Corporation, Tokyo, Japan) with an acceleration voltage of 3 kV, beam inclination angle of 5°, eccentric rotation of 5 mm, and rotation speed of 25 rpm for 5 min. The imaging was performed using a JEOL-7800F Scanning electron microscope (SEM) (JEOL Ltd., Tokyo, Japan), operated at 7 kV. Energy-dispersive X-ray spectroscopy (EDS) (Bruker Quantax, Bruker Nano GmbH, Berlin, Germany) was employed in order to determine the concentration of Ti, Zr and W. A sub-micron spatial resolution was required for the EDS measurements, and 7 kV was adopted as the accelerating voltage for analyzing the Ti-K, Zr-L, and W-M peaks. The resulting spatial resolution during the equilibrium phases is estimated to be about 200 nm, recorded using Monte Carlo electron trajectory simulations [20,21].

## 3. Results and Discussion

### 3.1. Final Microstructure and Properties

**Figure 1:**
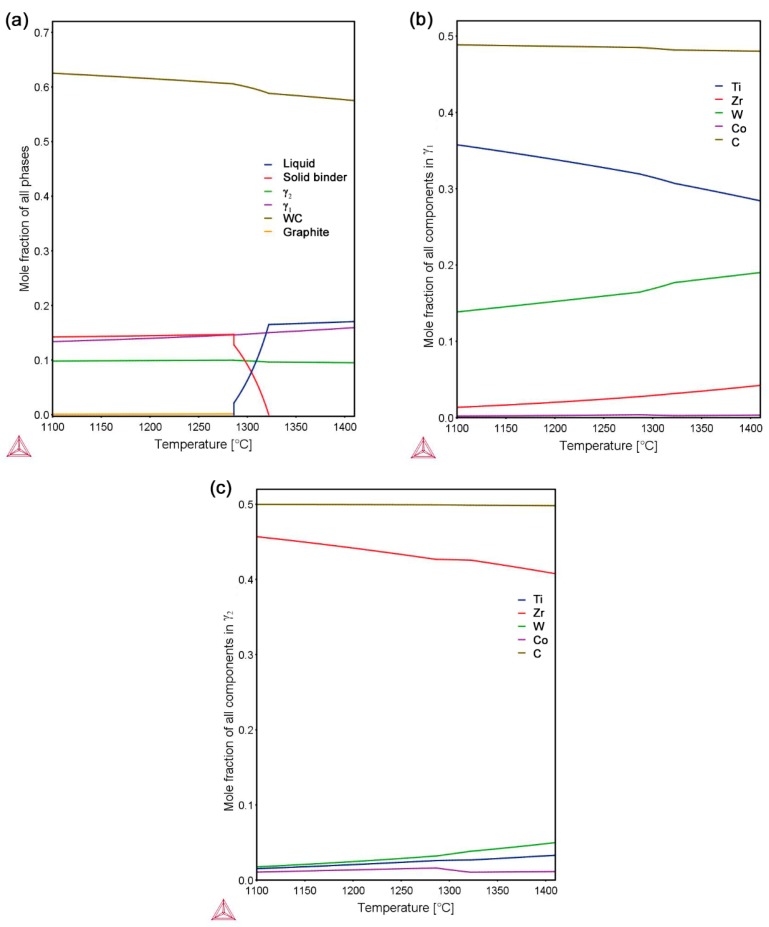
(**a**) Mole-fraction of stable phases in the WC-TiC-ZrC-Co system and equilibrium composition of (**b**) γ_1_ and (**c**) γ_2_ versus temperature predicted from thermodynamic calculations using Thermo-Calc and the TCFE8 database.

**Figure 2:**
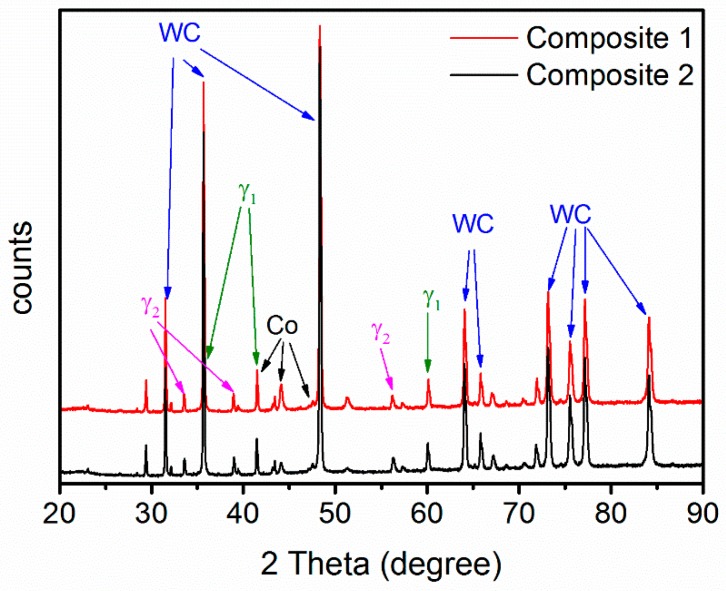
XRD patterns of composite 1 and 2 after sintering at 1410 °C for 1 h.

**Figure 3:**
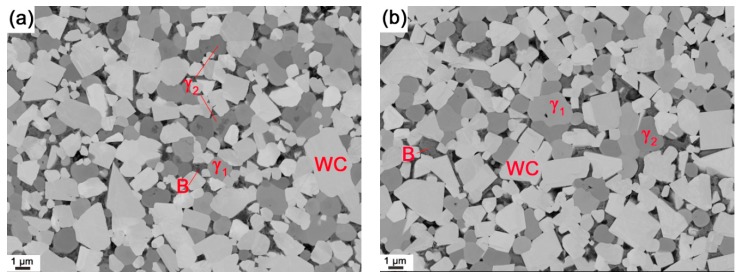
SEM backscattered electron micrographs of (**a**) composite 1 and (**b**) composite 2 after sintering at 1410 °C for 1 h. The equilibrium phases present in the final microstructure are indicated; the letter B refers to the binder phase.

The equilibrium phase fractions and compositions at different temperatures were calculated using Thermo-Calc (Thermo-Calc Software AB, Stockholm, Sweden) and the TCFE8 Steels/Fe-alloys database, version 8 (Thermo-Calc Software AB, Stockholm, Sweden) [22]. The results are presented in Figure 1. At a sintering temperature of 1410 °C, it is predicted that the equilibrium structure consists of four phases: liquid binder, WC, γ_1_, and γ_2_. This agrees with the results of the XRD measurements on composite 1 and 2 after sintering, presented in Figure 2. Four phases, corresponding to the binder and the three carbides, are identified in the diffraction patterns. WC is the dominant carbide phase, but small amounts of Ti-rich (Ti,W,Zr)C and Zr-rich (Zr,W,Ti)C, i.e., γ_1_ and γ_2_, are also present in both composites. The micrographs presented in Figure 3 show overviews of the sintered composites, and EDS analysis was used to identify each phase. As indicated in Figure 3, the white particles are WC, the light gray particles are γ_1_, and the dark gray particles are γ_2_. The black regions among the carbide particles are either binder phase or pores.

The carbide grain size after sintering is presented in Table 1, and it can be seen that the average grain size of WC in composite 1 is about 10% smaller than in composite 2. This is in agreement with the measured magnetic coercivity, which is inversely related to the WC grain size. Furthermore, the average grain size of both the γ_1_ and the γ_2_ phases is smaller in composite 1 than in composite 2. Therefore, the initial ternary carbide powder has a more limited grain growth of carbide grains in general. The hardness of composite 1 may also be slightly affected by its smaller grain size, but the measured difference in hardness is too low to draw any definitive conclusion. Composite 1 is pore-free after sintering, while composite 2 has a very low porosity.

### 3.2. Sintering Behavior

**Figure 4:**
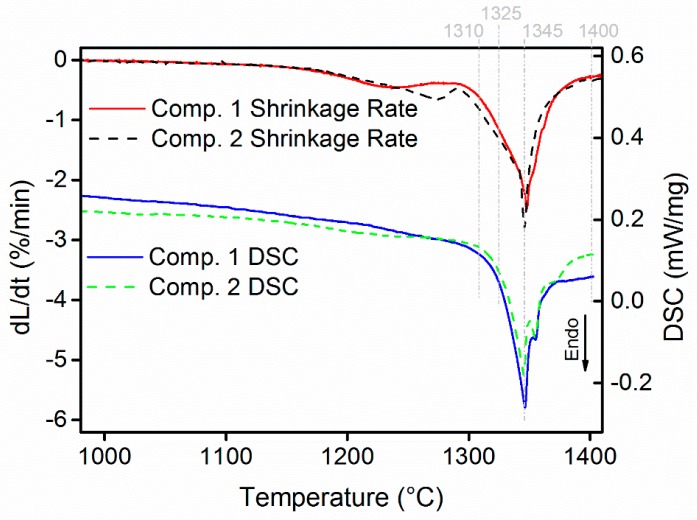
Shrinkage rates and differential scanning calorimetry (DSC) curves of composite 1 and 2. The temperatures indicated by gray dashed lines are selected for interrupted sintering.

According to the model by Petersson and Ågren [23,24], the shrinkage of cemented carbide during sintering can be divided into three stages. The first two correspond to solid-state shrinkage, but with different shrinkage rates, and the third is liquid-state shrinkage. Between the second and third stages, there is a region called the intermediate stage, during which the material is in both solid and liquid state. Applying the model to the dilatometry results for composite 1 illustrated in Figure 4, the second and third stages of shrinkage occur at 1180–1280 °C and 1348–1450 °C respectively, with a rather wide intermediate stage at 1281–1347 °C, during which the shrinkage rate increases sharply. Composite 2 exhibits a similar shrinkage behavior, except for the end point of the second stage shrinkage, which occurs at a slightly higher temperature, around 1290 °C; when compared with Petersson and Ågren’s model based on uniaxial viscosity [23], this indicates that the viscosity of composite 2 decreases at a slower rate than that of composite 1 in the second stage shrinkage. The total shrinkage is 18.2% and 19.9% for composite 1 and 2, respectively. As can be seen from the DSC data in the bottom section of Figure 4, the onset melting in both composites, estimated to be 1322 °C by using the common tangent method, is in reasonable agreement with the initiation of the aforementioned intermediate shrinkage stage. However, the change in the curvature of the DSC curves before reaching 1322 °C indicates that a low fraction of molten binder already exist. The major endothermic reaction peak is found at 1345 °C in both composites, while a minor additional endothermic peak is found at 1355 °C for composite 2.

### 3.3. Microstructural Evolution during Sintering

#### 3.3.1. **γ** Phase in Composite 1

**Figure 5:**
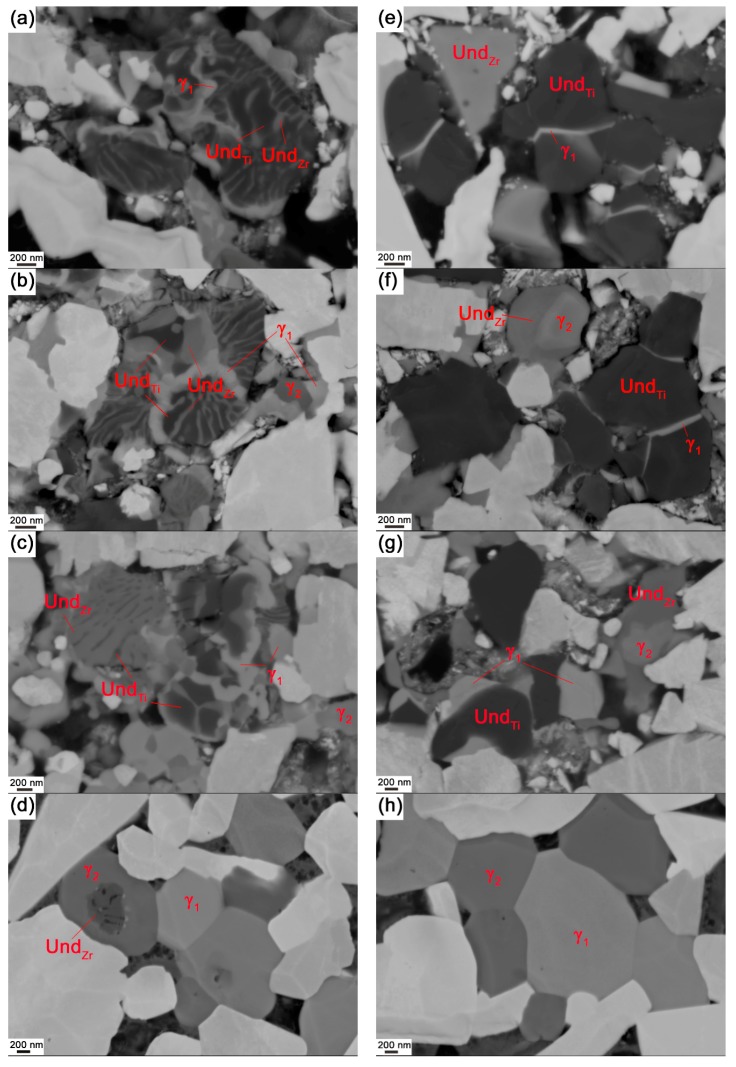
SEM backscattered electron micrographs of cubic carbide phases in composite 1 interruptedly sintered at (**a**) 1310 °C; (**b**) 1325 °C; (**c**) 1345 °C; (**d**) fully sintered; and in composite 2 interruptedly sintered at (**e**) 1310 °C; (**f**) 1325 °C; (**g**) 1345 °C; (**h**) fully sintered. Und_Ti_ and Und_Zr_ represent undissolved Ti-rich and Zr-rich original cubic carbide phases respectively.

**Figure 6:**
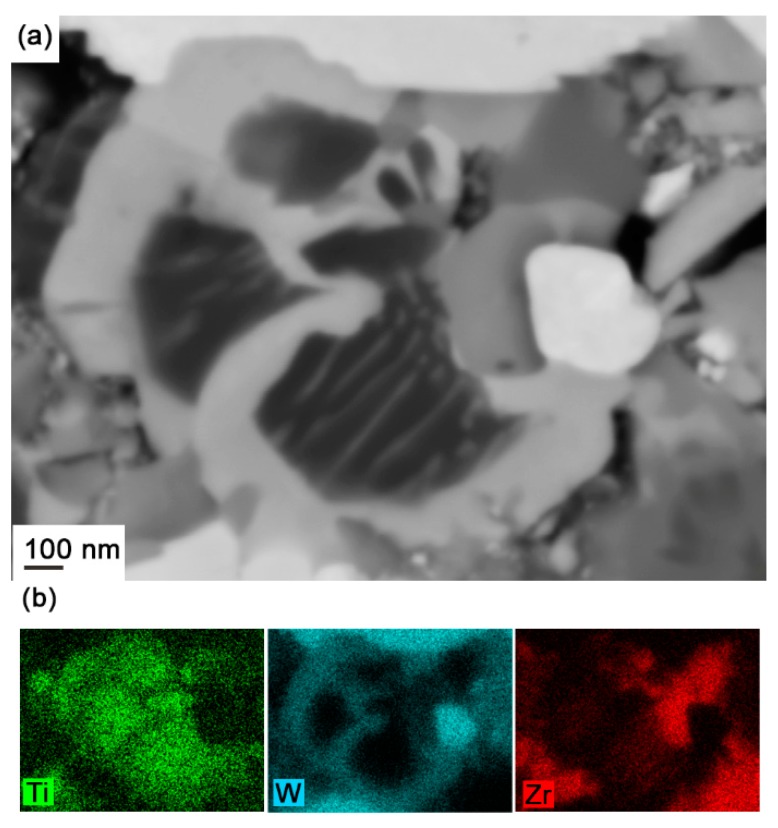
(**a**) SEM backscattered electron micrograph and (**b**) energy dispersive X-ray spectroscopy (EDS) map of cubic carbide phases in composite 1 interruptedly sintered at 1345 °C.

Figure 5a shows the microstructure of composite 1 interruptedly sintered at 1310 °C. This temperature is lower than the melting point as determined by DSC (1322 °C), but as previously mentioned the DSC curve indicates that partial melting of the binder would have already occurred. As can be seen in Figure 5a, a lamellae structure has formed, and, moreover, light gray strips are found to precipitate along (Ti,Zr)C grains. When the temperature increases, more light gray strips are forming and thickening, and they completely encircle the (Ti,Zr)C lamellae in the center of the particles, see Figure 5b,c. According to the EDS mapping analysis in Figure 6, these strips contain large amounts of W and further EDS point analysis revealed that the light gray strips are (Ti,Zr,W)C, i.e., γ_1_, whose composition at different temperatures is measured and listed in Table 2. The γ_1_ strips were observed to already form after interrupted sintering at 1235 °C, i.e., prior to melting, and hence the dissolution of metallic atoms, i.e., W, Ti, and Zr, and re-precipitation, already occurs during the solid-state sintering stage. A similar case has been reported in Zackrisson et al. [25].

In addition to the light gray strips along (Ti,Zr)C grains, γ_1_ phase is also present in the gaps between carbide grains as individual particles. These particles can be observed from a temperature of 1325 °C and higher, see Figure 5b,c, during which significant melting occurs and liquid binder phase forms, allowing faster dissolution and re-precipitation of metallic atom (W, Ti, and Zr) [4]. Moreover, some liquid binder phase flows towards interparticle spaces to fill the pores and γ_1_ phase precipitates upon cooling, following the supersaturated binder phase, as individual grains.

Finally, both individual γ_1_ particles and strips evolve to become homogeneous γ_1_ grains (see Figure 5d). This indicates that those areas of (Ti,Zr)C phase encircled by light gray γ_1_ rims are dissolved during the later stages of sintering.

**Figure 7:**
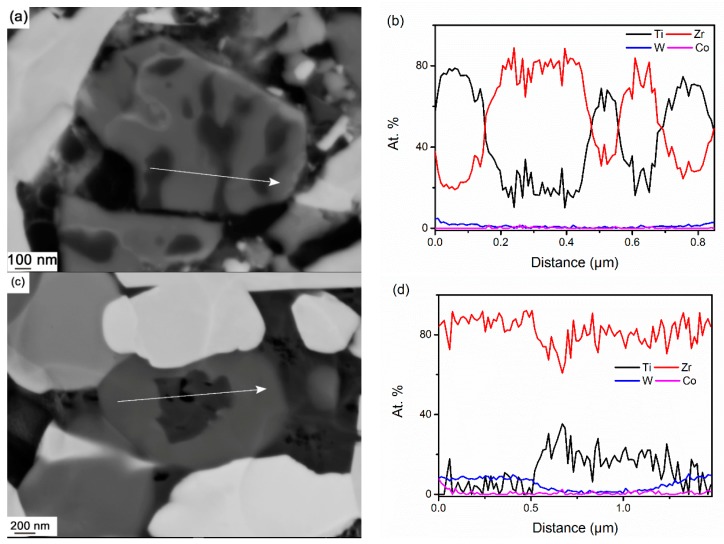
EDS line scan (**a**,**b**) across a (Ti,Zr)C lamellae in composite 1 interruptedly sintered at 1310 °C, (**c**,**d**) through γ_2_ with dark core in fully sintered composite 1.

When considering the other γ-phase, individual γ_2_ particles have been observed after 1325 °C, see Figure 5b,c; no γ_2_ phase has been observed inside original (Ti,Zr)C particles up to 1345 °C. A small number of γ_2_ grains are found to contain darker cores after full sintering, and the composition of these is found to be close to residual (Ti,Zr)C phase, see Figure 7c,d. This indicates that part of the (Ti,Zr)C phase has not been dissolved during sintering.

The composition of γ phases in composite 1 at different temperatures is measured and listed in Table 2. As can be seen in the γ_1_ phase, the solubility of Ti decreases, whilst that of W increases, with rising temperature, which is in good agreement with the Thermo-Calc calculation shown in Figure 1b. The rather small variation in the solubility of Zr cannot be concluded using the present EDS measurement. The γ_2_ grains in composite 1 between 1325 °C and 1345 °C are usually around 100 nm, which is beyond the EDS resolution; due to the influence of surrounding phases, the Zr content is underestimated in the present measurement. Therefore, the variation in composition cannot be concluded. According to the Thermo-Calc calculation, Figure 1c, the solubility of Zr in γ_2_ phase decreases, whereas the solubility of W and Ti increases, with rising temperature.

#### 3.3.2. **γ** Phase in Composite 2

In Composite 2, the γ-phase is principally found to precipitate on original TiC and ZrC particles. As can be seen in Figure 5e, γ_1_ light gray strips, similar to those in composite 1, start to precipitate along TiC grains before melting. These strips are thickening, as dissolution and re-precipitation continue, see Figure 5f,g. Finally, all of the TiC grains are dissolved, and the resultant γ_1_ grains are generally homogeneous after full sintering, see Figure 5h.

The γ_2_ phase is found to precipitate on ZrC grains after 1325 °C, but instead of enclosing the original grains, the new phase directly grows into the ZrC grain, see Figure 5f,g. The fact that the γ_2_ phase develops in this way is probably due to similarities in the composition and the lattice between the ZrC and γ_2_ phase with a composition of about (Zr_0.85_W_0.1_Ti_0.05_)C. In order to reach composition equilibrium, only a small number of Zr atoms in the original ZrC lattice needs to be substituted by W or Ti. Furthermore, both ZrC and γ_2_ lattices are FCC with rather similar lattice parameters of 4.69 and 4.62 Å, according to the XRD measurement. Therefore, the transformation proceeds with rapid kinetics in this case. In the final sintered composite, all of the ZrC phase has been replaced by a rather homogeneous γ_2_ phase, see Figure 5h.

Similarly, as in composite 1, γ_1_ precipitates earlier than γ_2_, and the most probable reason for this is the limited solubility of Zr in the solid binder phase. Therefore, γ_2_ is only capable of forming after the binder phase has melted, since it can then dissolve more Zr and γ_2_ can re-precipitate.

The measured composition of γ phases at different temperatures in composite 2 corresponds to that in composite 1, see Table 2, except for the γ_2_ phase at 1325 °C. The reason for this, as mentioned above, is most likely to be that the Zr content of γ_2_ phase in composite 1 has been underestimated due to the small grain size.

#### 3.3.3. Phase Separation of (Ti,Zr)C

The original cubic carbide in composite 1 is a powder of the ternary mixed carbide (Ti,Zr)C, with an overall composition around Ti_0.5_Zr_0.5_C. Detailed information can be seen in our previous work [26].

The originally homogeneous (Ti,Zr)C grains decompose into Ti-rich (black) and Zr-rich (gray) lamellae during solid-state sintering, due to the instability of the mixed carbide within the miscibility gap. The first observations of this decomposition were found after interrupted sintering at 1185 °C, but the decomposition becomes more evident after sintering at higher temperatures, and it is exemplified after interrupted sintering at 1310 °C in Figure 5a and Figure 7a,b. It should be noted that, in this condition, part of the binder phase may already have become molten. This decomposition is similar to previous observations in the (Ti,Zr)C ternary system [13,26], which resembles discontinuous precipitation (DP), rather than spinodal decomposition [26], and it was also found that the DP reaction proceeded very slowly. However, the reaction proceeds much faster in the present case. It is known that Co can spread out and wet refractory particles, e.g., WC and (Ti,Zr)C, during solid-state sintering [4,27]. Following this, it can be speculated whether the presence of Co, and perhaps also W and C, could increase the diffusivity of Ti and Zr along the former grain boundaries and phase boundaries between the lamellae, thus increasing the rate of the DP reaction. In any case, the dramatic change in the kinetics of the reaction is still somewhat surprising and needs further investigation.

As discussed in Section 3.3.1 and Section 3.3.2, the precipitation site of γ phases is highly dependent on original cubic carbide particles. In composite 2 (Figure 5e–h), γ_1_ phase prefers to precipitate on original TiC grains, due to their similar composition, and the same is true for γ_2_ phase on ZrC grains; in general, each γ grain precipitates and grows independently. Comparably, in composite 1, it is reasonable to deduce that γ_1_ and γ_2_ phases tend to precipitate on Ti-rich and Zr-rich carbides, respectively. On the other hand, because of the proximity between the alternative nano-scale lamellae, the dissolution process of Ti-rich and Zr-rich carbide phase would mutually interfere with each other, affecting the re-precipitation of the two γ phases. The results illustrate that γ_1_ precipitates easily on (Ti,Zr)C grains, consuming a large amount of (Ti,Zr)C phase, while γ_2_ phase was not observed on (Ti,Zr)C grains up to 1345 °C, see Figure 5a–c. This is probably because the dissolution of Zr-rich (Ti,Zr)C phase has been restrained. Further evidence can be seen in the final microstructure, in which remnant Zr-rich (Ti,Zr)C is found as darker cores in some γ_2_ grains, see Figure 7c,d. It is clear that Ti-rich lamellae dissolve preferentially in the binder phase with respect to Zr-rich lamellae. This could be due to the higher instability of Ti-rich (Ti,Zr)C phase, which arises from the large difference in composition between Ti-rich lamellae, (Ti_0.82_W_0.03_Zr_0.15_)C, and equilibrium γ_1_ phase, (Ti_0.64_W_0.29_Zr_0.07_)C.

The different precipitation reactions is most probably responsible for the difference in carbide grain size. However, the detailed mechanism for this is not understood, and further investigation is required.

## 4. Conclusions

- WC-(Ti,Zr)C-Co and WC-TiC-ZrC-Co have been liquid phase sintered at 1410 °C in the present work. Both composites present similar shrinkage behavior, and WC-(Ti,Zr)C-Co has a lower total shrinkage, of 18.2%; both composites have a similar melting temperature of about 1322 °C; after sintering, both composites contain two γ phases—(Ti,W,Zr)C and (Zr,W,Ti)C cubic carbides.
- After full sintering the grain size of both WC and cubic carbides in WC-(Ti,Zr)C-Co is around 10% smaller than that in WC-TiC-ZrC-Co. The ternary carbide powder thus has a grain growth inhibiting effect, but only a small difference has been found in the measured hardness.
- During interrupted sintering, γ_1_ re-precipitates before binder melting, while γ_2_ re-precipitates directly after binder melting in both composites. The composition of γ phases measured by EDS is in good agreement with the thermodynamic calculations.
- In WC-TiC-ZrC-Co, γ_1_ and γ_2_ phases mainly re-precipitate on original TiC and ZrC grains respectively; in WC-(Ti,Zr)C-Co, except on the original (Ti,Zr)C grains, considerable amounts of γ_1_ and γ_2_ phases also re-precipitate as individual grains in the interparticle spaces.
- In WC-(Ti,Zr)C-Co, (Ti,Zr)C phase decomposes into Ti-rich and Zr-rich nano-scale lamellae at a rapid rate during solid-state sintering; Ti-rich lamellae dissolve at a much faster rate than Zr-rich lamellae in liquid binder phase, and a small amount of Zr-rich (Ti,Zr)C phase is retained as darker cores in γ_2_ particles after full sintering.

## Figures and Tables

**Table 1:** Grain size and properties of sintered composites.

Composite	d_WC_ (µm)	d_γ1_ (µm)	d_γ2_ (µm)	H_c_ (kA/m)	RMS	Hardness (HV3)	Porosity	Mass Loss (%)
1	1.11	1.04	0.89	11.64	0.96	1448	A00B00C00	2.10
2	1.21	1.19	1.03	10.73	0.97	1443	A04B00C00	2.29

d_WC_, d_γ1_ and d_γ2_—mean grain size of WC, γ_1_ and γ_2_ phases; H_c_—magnetic coercivity; RMS—relative magnetic saturation; Mass loss during sintering = ((Pressed weight − Sintered weight)/Pressed Weight) × 100.

**Table 2:** Concentration of metallic components in the cubic carbide phases at different temperatures, measured by EDS (at %).

Status	Comp.	Undissolved Cubic Carbides						Re-Precipitated Cubic Carbides					
		Ti-Rich			Zr-Rich			γ_1_			γ_2_		
		Ti	Zr	W	Ti	Zr	W	Ti	Zr	W	Ti	Zr	W
1310 °C IS	1	80	19	1	18	80	2	73	10	17	-		
	2	100	0	0	0	100	0	76	3	21	-		
1325 °C IS	1	89	9	2	23	74	3	63	13	24	15	73	12
	2	99	0	1	0	99	1	67	6	27	5	85	10
1345 °C IS	1	82	15	3	15	78	7	64	7	29	9	80	11
	2	100	0	0	1	97	2	65	6	29	7	80	13
1400 °C IS	1	89	5	6	13	80	7	61	8	31	6	85	9
	2	100	0	0	-			61	8	31	6	83	11
1410 °C FS	1	-			23	74	3	62	6	32	6	85	9
	2	-			-			60	7	33	6	83	11

IS and FS represent interrupted and full sintering respectively. Comp. represents composition. The Co concentration is lower than 1 at. % in all the cubic carbide phases, and it is not included in this table.
